# Rural response system to prevent violence against women: methodology for a community randomised controlled trial in the central region of Ghana

**DOI:** 10.1080/16549716.2019.1612604

**Published:** 2019-05-28

**Authors:** Adolphina A. Addo-Lartey, Deda Ogum Alangea, Yandisa Sikweyiya, Esnat D. Chirwa, Dorcas Coker-Appiah, Rachel Jewkes, Richard M. K. Adanu

**Affiliations:** a Department of Epidemiology and Disease Control, School of Public Health, University of Ghana, Accra, Ghana; b Department of Population, Family and Reproductive Health, School of Public Health, University of Ghana, Accra, Ghana; c Gender and Health Research Unit, South African Medical Research Council, Pretoria, South Africa; d Gender Studies and Human Rights Documentation Centre, Accra, Ghana

**Keywords:** Intimate partner violence, gender-based violence, community intervention, impact assessment, community-based action teams, cluster randomised controlled trial

## Abstract

Violence against women (VAW) is common in Ghana, with nation-wide surveys reporting high prevalence of intimate partner violence (IPV) (physical, sexual and/or emotional violence). Our trial assesses the community level impact of the Rural Response System which uses Community-Based Action Teams ‘COMBAT’ for preventing VAW in Ghana. This study is a mixed method unmatched cluster randomised controlled trial and includes rural (n = 23), peri-urban (n = 7) and urban (n = 10) communities in four districts of the Central Region, Ghana. The trial will last three years with one baseline survey, one impact assessment and a qualitative baseline, midpoint and endline evaluation. A total of 40 localities were selected to serve as clusters (20 per trial arm) with about 82 households per cluster recruited at baseline. The same number will be recruited post-intervention. Adult women (18 to 49 years) and men (≥ 18 years) were drawn from different localities. Sampling of households within a community was random and done using a computerised system. In each selected household, one female or male resident was invited to participate. Individuals are eligible for inclusion in the study if they usually live (sleep and eat) in the household, have lived in the community for at least a year, and are between the ages of 18-to-49 years old. Our impact assessment component will compare past 12 months incidence of IPV (i.e. IPV experiences for women and perpetration of physical and/or sexual IPV for men) between arms in the trial. The implementation of this community trial comes at an opportune time when evidence on the effectiveness of a targeted VAW intervention in the Ghanaian society is needed to inform the development of national policies for preventing VAW. Our progressive research approach using a mixed method design will further extend knowledge globally on a multifaceted intervention to reduce the incidence of intimate partner violence in a developing country.

## Background

Violence against women (VAW) is a global public health burden that has significant consequences for women’s mental and physical well-being, including their reproductive and sexual health [1–5]. Intimate partner violence (IPV), which describes physical, or emotional, or sexual assault, or both, of a spouse or sexual partner, is a common form of VAW in many parts of the world [6], including Ghana [4,5,7–9]. A national study in 1998 revealed that 33% of Ghanaian women experienced physical violence at the hands of their current or previous partners, 29% had their first experience of sexual intercourse by force, whilst 33% of the women had been touched inappropriately against their will [7]. Findings from the Ghana Demographic Health Survey (GDHS) further indicate that among women aged 15–49 years, 36.6% reported experience of physical violence since age 15, 17.2% reported experience of violence in the 12 months preceding the survey and 18.8% reported lifetime experience of sexual violence [8]. Among ever-married women, the GDHS data showed that 34.9% have experienced some form of violence (physical, sexual, or emotional) by a husband or partner in the 12 months preceding the survey [8].

Estimates of IPV prevalence in Ghana have been steadily increasing over the years. In 2011, the UN Women’s violence against women prevalence survey in Ghana reported that between 13% and 61% of ever partnered women in Ghana have experienced a spectrum of lifetime physical violence [9]. While the main perpetrators of physical and sexual violence against women tend to be current or previous husbands and partners; strangers, teachers, schoolboys, other family members and acquaintances may also be perpetrators of physical and sexual violence [4,8]. Violence against women in Ghana is influenced by several factors, including social, economic and legal elements [3,5]. Existing data from the Ghana AIDS Commission (GAC) suggest that Ghanaian women often experience relative powerlessness, compared with men, because of poor economic empowerment and negative social norms [10]. As a result, they are often subject to the will of their partners and husbands. This powerlessness, along with limited life choices, makes it difficult to decline sexual advances without facing coercion or violence [10].

Although there have been very few nation-wide VAW prevalence studies in Ghana leading to limited empirical evidence on which prevention strategies could be based, VAW continues to be a growing public health issue in Ghana [7–10]. The absence of data pertaining to a long-term impact assessment of VAW interventions is therefore a key gap in VAW research in Ghana. For instance, even though the Rural Response System (RRS) which uses a community response model (Community-Based Action Teams ‘COMBAT’) for preventing VAW was developed in 2002 and subsequently piloted for two years in three communities (located in the Eastern, Ashanti and Upper East Regions of Ghana), an impact evaluation of the intervention was not done. After the first two years of implementation, the intervention was scaled up in 15 other communities for three more years (2005 to 2008), in the Eastern region, Ashanti, Upper East and Upper West Regions of Ghana. However, there has been no follow up in these beneficiary communities since 2008 to assess the long-term impact of the RRS [7]. An integral component for the success of the RRS is stakeholder buy-in, hence training sessions on VAW and support systems were held for state agencies mandated to handle VAW cases (i.e. the Police/Domestic Violence and Victim Support Unit (DOVVSU), Department of Social Welfare (DSW), Commission on Human rights and Administrative Justice (CHRAJ) and Ghana Health Service (GHS). Unfortunately, there has been no assessment of the district level capacity-building training sessions undertaken for staff of the state agencies. This trial assesses the impact of the Rural Response System intervention delivered to all adult women and men living in selected districts in the Central Region of Ghana.

## Methods

### Aim and objectives

The overall aim of this trial is to evaluate a multi-faced community intervention to reduce the incidence of IPV in selected communities in Ghana. Regarding the RRS model, the specific objectives of the impact assessment will be as follows:

- To evaluate the impact of the RRS model in preventing/reducing VAW
- To determine whether RRS intervention is effective in enabling women in to reduce their exposure to IPV and men to reduce their perpetration of IPV
- To assess the extent to which RRS has changed individual attitudes about gender inequality
- To determine if the RRS model changes discriminatory social norms and gender roles which consider VAW acceptable
- To examine state institutional response to reported cases of VAW
- To assess whether the RRS model facilitated a shift in power relations between men and women in the household/community to become more equitable

### Study design

The study is a mixed method unmatched cluster randomised controlled trial with two arms. The intervention arm receives the rural response system (RRS) intervention for preventing VAW for 18 months while the control arm received no intervention. Owing to the nature of the intervention, participants will not be blind to their trial arm. Sampling of participants will follow a repeat-cross-sectional community survey. The trial is being conducted over 36 months with one quantitative and qualitative baseline assessment (2016) and one qualitative midpoint evaluation (2017). The impact assessment components (both quantitative and qualitative) will occur at 24 months post-baseline (2018). Our trial protocol has been registered and is available on ClinicalTrials.gov (NCT03237585). We did not make any important changes to the trial design after trial commencement.

#### Study setting

**Figure 1.**
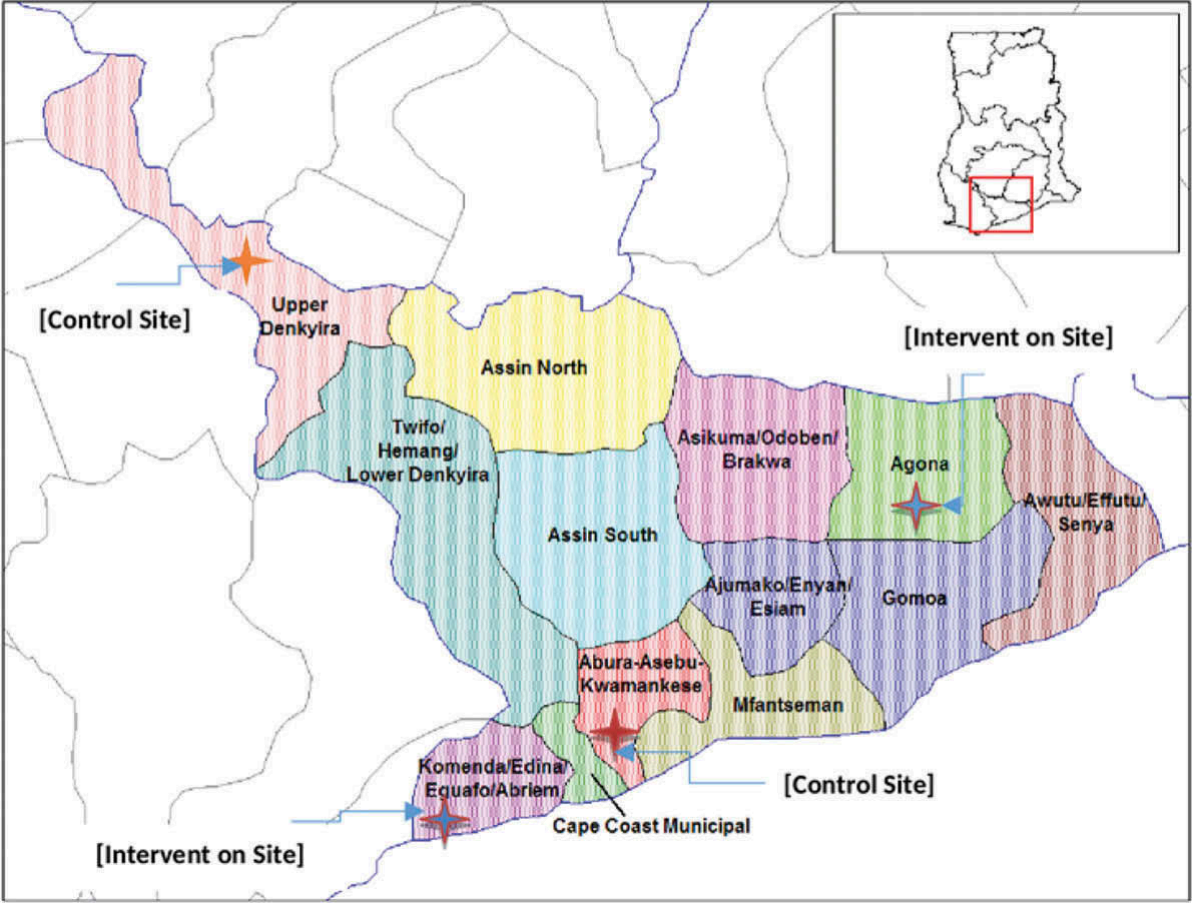
Location of the 4 study sites (Districts) in the central region of Ghana.

This trial is being carried out in four districts located in the Central Region of Ghana. Two districts are along the Coast while the other two are inland districts. Figure 1 shows the location of the Central Region on the map of Ghana and the location of the study sites (districts) relative to each other. The Central Region occupies an area of approximately 9,800 square kilometres, which is about 6.6% of the land area of Ghana [8,11]. About 63% of the region is rural and the population was estimated at 2,413,050 for the year 2013 with an annual growth rate of 3.1% and a population density of about 215 inhabitants per square kilometre [8,11]. Adult literacy rates in the region is around 50%, with more men literate (69.8%) than women (46.3%) in the region [11]. The region is predominantly Akan speaking (82.0%) with Fante being the indigenous dialect of most districts in the region [11]. Unemployment rate is 8.0% which is 2.4% lower than the national average [11]. The unemployment rate among women is 8.2% and this is about 0.4% higher than in men in almost all the districts in the Ghana. Agriculture is the main occupation and employs more than two-thirds of the work force in many districts. Cocoa and oil palm production are concentrated mainly in the inland districts while pineapple, grain production, and fishing are concentrated mainly in the coastal districts [11].

#### Selection and randomisation of study sites

**Figure 2.**
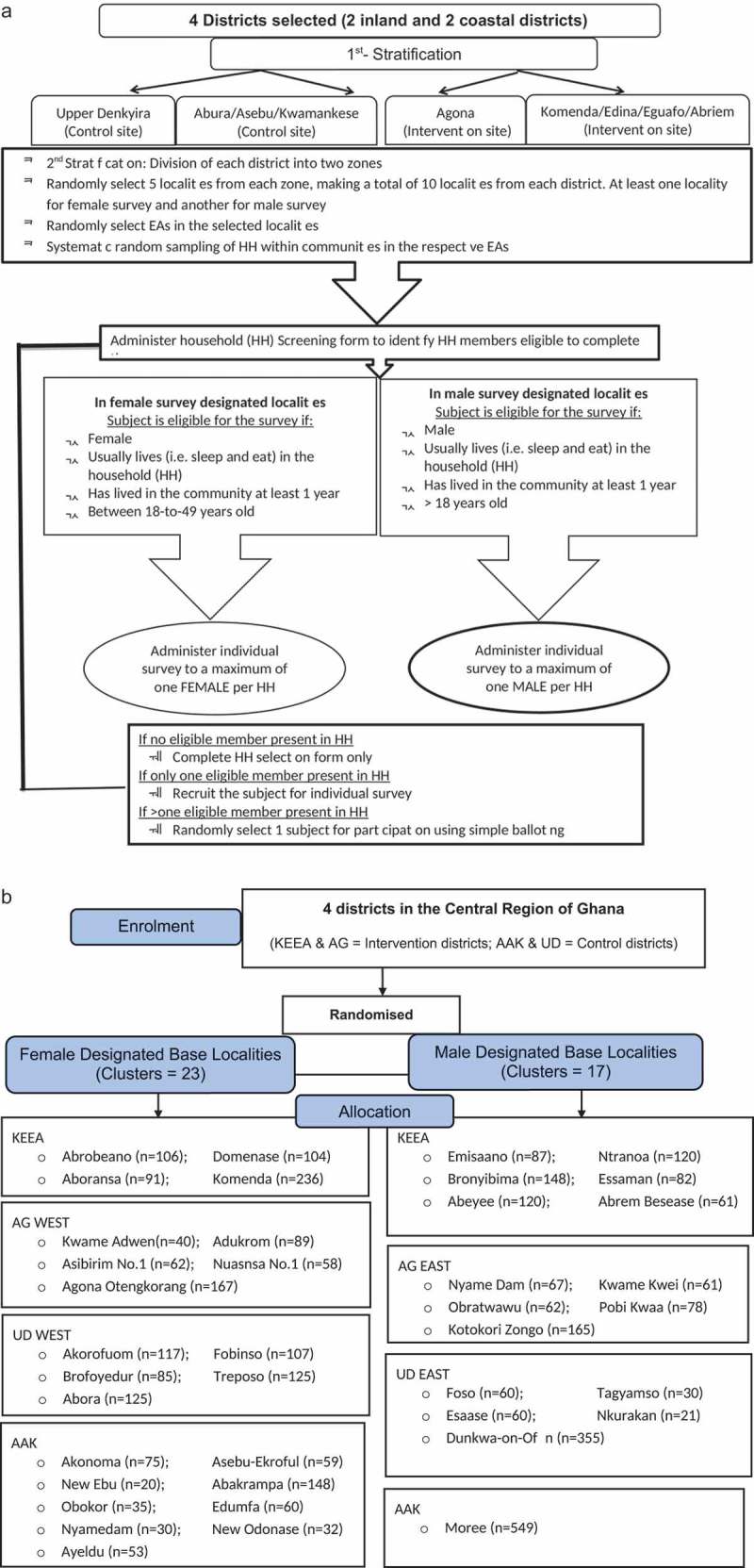
(a) Sampling strategy at baseline and post-intervention. (b) CONSORT diagram for participant selection in the localities.

The selection of intervention and control districts was done using a census map of the Central Region that showed Inland and Coastal districts. After excluding some districts because of previous VAW-related intervention activities conducted by the Gender Studies and Human Rights Documentation Centre (Gender Centre; two inland and two coastal districts were then purposively selected as study sites. Designated sites are separated from each other by a geographical buffer (at least one district wide) to reduce the possibility of intervention diffusing into control districts (Figure 1). To randomise the districts, the names of the two Inland districts were written on a piece of paper and placed in a bag. One paper was blindly drawn from the bag and the selected district was assigned as an intervention district, while the other was designated as the control district. Clusters in the Ghana Demographic and Health Survey (DHS) are census listed localities with each locality comprising one or more Enumeration Areas (EAs). Within each selected district, a list of localities was obtained from the Ghana Statistical Service (GSS). All localities were allocated numbers, placed in a bag and blindly drawn. A total of 40 localities, (10 per district) were selected to serve as clusters. Since localities comprise several EAs, there was a second layer of stratification such that within each district, women and men participants were drawn from different localities(Figures 2a & b).

#### Recruitment and inclusion/exclusion criteria

We recruited adult women (ages 18 to 49 years) and adult men (≥ 18 years) in the Central Region of Ghana. A multistage stratified cluster random sampling process was used to sample households (individuals) within selected communities at baseline and the same procedure will be repeated at endline. An overview of the randomisation and recruitment process is shown in Figures 2(a & b). In both Intervention and control districts, about 82 households were selected from 10 localities in each district. Sampling of households within a community was random and was done using a computerised system. In each selected household, one female or male subject was invited to participate in the trial. Individuals are eligible for inclusion in the study if they usually live (sleep and eat) in the household and lived in the community for at least a year. A limit of one respondent per household was set out of consideration for safety and confidentiality. All selected participants must be able to communicate in the main languages of the study (English, Twi, and Fante) and not be suffering from a mental deficit (learning difficulty, mental illness or substance abuse) which would impair their ability to consent to participation in the trial. E.g. individuals with psychosis cannot consent for research participation, but those with other mental illnesses, such as post-traumatic stress disorder or depression, would not be excluded. Furthermore, having consumed alcohol does not exclude a person from participating in the trial, it is only being currently drunk/drugged that stops consent being given. This inclusion/exclusion criteria are important in order not to bias the sample away from people who have experienced violence who may have substance abuse and other mental health problems.

### Sample size estimation

Our sample size calculation for a community RCT was defined at two levels, i.e. the level of randomisation – number of clusters randomised, and the level of data collection – number of individuals surveyed in each household. Using the method of Hayes and Bennet [12], we estimated sample size (i.e. the number of clusters required in each arm of treatment) for an unmatched cluster-randomised control trial. The calculation made the following assumptions. The proportion in the population that experience any form of IPV (emotional, physical, sexual violence, or all three forms). This estimate is based on national-level data from the 2008 GDHS which indicates that among ever-married women age 15–49 years, 34.9% have experienced violence (any form) committed by their husband/partner in the 12 months preceding the GDHS survey [8]. We powered the study to detect a 30% reduction in violence in the intervention arms and took alpha (ɑ) = 0.05 (two-sided), set power at 90% and estimated the coefficient of variation (k) between clusters (localities) for the outcome measure to be k = 0.20 based on district-level census data from the Central Region of Ghana [11]. We planned to oversample by 15% to allow for incomplete questionnaires. Based on these calculations, a minimum sample size of 820 women and 820 men from 40 clusters per trial arm were sampled at baseline and the same numbers will be sampled again in the post-intervention survey. This would imply 90% power to detect a significant change at the 5% level with a 20% reduction in IPV incidence.

### Intervention description

#### The rural response system (RRS)

The RRS was developed by the Gender Studies and Human Rights documentation Centre (Gender Centre) in 2002 to address some of the key findings from its 1998 study on VAW and children in Ghana [7]. The research also showed that VAW was not seen as a crime but was viewed as a private matter that should be taken care of within the family [7]. Owing to this outlook, interventions were an intrusion into a private situation. This societal view consequently inhibited women from talking about their experiences of violence. When women chose to talk about their experiences of violence, they preferred to report informally to family, friends or members of the community [7]. The 1998 study by Gender Centre also revealed a lack of support for victims of violence; such that when women did report violence committed against them, state agencies were the least likely venue for them to report [7]. The women believed that the attitude of state agency personnel, with the same biases as the society in which they operate, tended to reflect in how they responded to cases of violence [7]. While this lack of support was evident throughout the country, it was more so in the rural communities. The RRS was subsequently developed by the Gender Centre as a strategy to deal with four major problem areas prioritised from the research findings [7]. These were (i). The poor state/institutional response to VAW, with frequent patterns of victim blaming, referring reported cases back to family and state agency personnel and society in general trivialising the issue; (ii). The high degree of tolerance of VAW in Ghanaian society, perpetuated by strong perceptions that domestic violence, i.e. violence that occurs in the home and in intimate relationships – is a private/family matter and not a serious crime; (iii). General confusion about what constitutes violence and ignorance about the causes, consequences and mechanisms that perpetuate VAW; and finally, (iv). Isolation of rural women and women’s expressed dissatisfaction with the assistance and support they received when they reported VAW cases.

**Figure 3.**
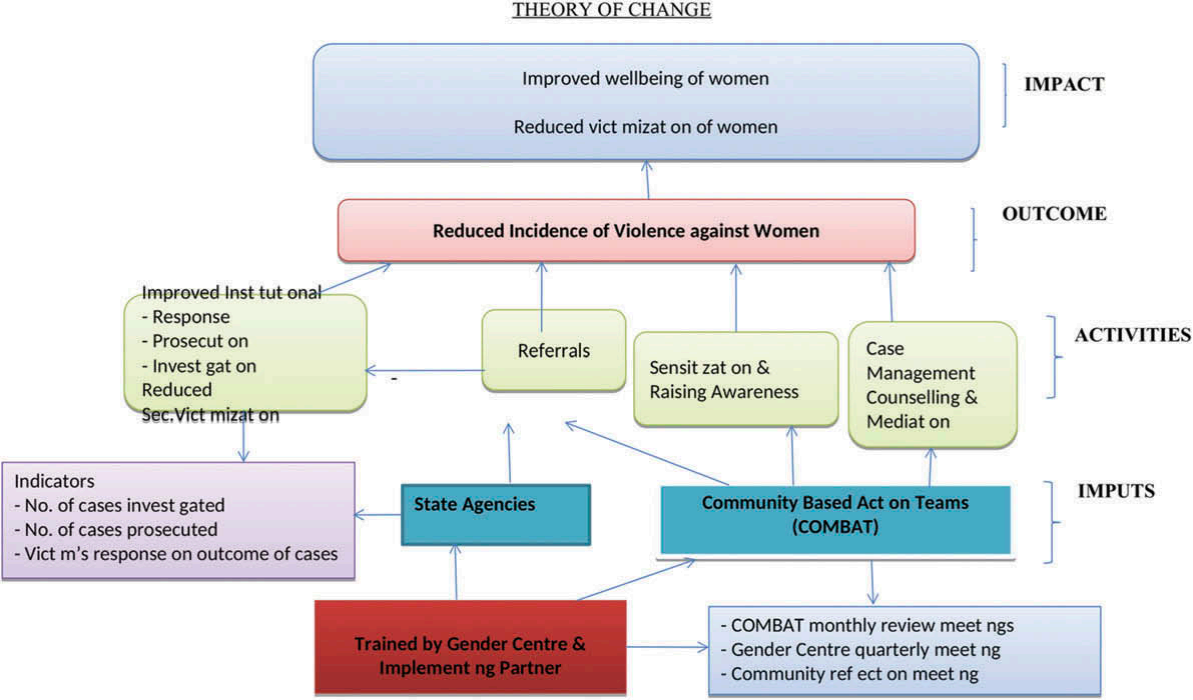
Intervention logic model to reduce incidence of violence against women in Ghana.

While the RRS intervention model was developed to respond to the problem areas prioritised from the research findings, it was inspired by the City of Duluth’s Coordinated Community Response (CCR) to Domestic Violence ‘The Duluth Model’ [13]. At the heart of the CCR is a shared understanding by all state and non-state partners involved that VAW women is a crime and a human rights violation and that the response to it must prioritise the safety and autonomy of the survivor. The RRS intervention is comprehensively described in the Gender Centre publication titled ‘A Guide to Developing a Community Response to Violence against women in Ghana’ [7]. Figure 3 shows the intervention logic model guiding our intervention delivery. As part of the RRS system, community-based action teams (COMBAT) are selected by community members and trained by the Gender Centre to create awareness on gender-based violence (GBV) and provide support to victims who report to them. The RRS operates on the theory that progressive change does not happen overnight. Ingrained norms and learned behaviours need sustained and cumulative interventions over long periods of time to deliver results. Representatives from State Agencies are also trained to aid them to respond effectively to cases that are brought before them. The overall result intended is a reduction of IPV, characterised by the improved well-being of women and men and the reduced victimisation of women in Ghana.

#### Community based action teams (COMBAT)

The RRS is stressed as a community initiative facilitated by the Gender Centre to foster community ownership. The selection of the COMBAT, the criteria for the selection of COMBAT and their roles are done by the community members with the Gender Centre serving only as a facilitator in this process. A COMBAT should be somebody who can keep confidences; that he/she is not known to be violent himself or herself; that he/she is somebody well respected in the community; and that he/she has the spirit of volunteerism and would commit to the project. It is a requirement that COMBAT members in a given community are composed equally of men and women. Whilst literacy is not a requirement for membership in the COMBAT, it is important to have at least one literate member to ensure that there is somebody available to handle the required documentation. Again, while no age limit has been set, the average age of members is generally about 40 years. Depending on the size of the community, the COMBAT membership range between 6 and 10.

#### Training of COMBAT

Once the community has accepted the concept of the RRS and has selected the COMBAT, the process of capacity building begins. The Gender Centre has developed a comprehensive training manual on violence which is used for these trainings and the training is carried out by seasoned facilitators using adult training methodologies. Initially, the COMBAT are given training on VAW, counselling and conflict resolution where they are taught to make available all options to the victim and empower her to take her own decision. COMBAT are made to understand that their role is limited to passing information and that it is the victim who must always take the decision. The in-depth training on VAW focuses on defining violence; explaining violence – including causes and factors such as gender roles and patriarchy, forms and types of violence, impact of violence, and laws in Ghana that deal with violence (Appendix 1). During the five-day training, several exercises are undertaken that allows the trainees to examine and interrogate their own biases and understanding and social norms. Since COMBAT are volunteers, they receive no remuneration from the Gender Centre for their work in the communities. However, refreshments are provided to the COMBAT during all training workshops and meetings.

#### Training of other stakeholders/state agencies

One of the key elements of the RRS is establishing linkages with those state agencies that in one way or the other encounter victims of violence or abuse. Training programs are directed at increasing the capacity of organisations, agencies and individuals to be able to respond to reported and suspected cases of VAW and children. These agencies and organisations include the Police/Domestic Violence and Victim Support Unit (DOVVSU), Department of Social Welfare (DSW), Commission on Human rights and Administrative Justice (CHRAJ) and Ghana Health Service (GHS). The staffs of these agencies are also given the same in-depth training on VAW as the COMBAT.

#### Role of COMBAT in the community

The role of COMBAT is clearly defined and can be summed up as sensitising community on issues on VAW by creating awareness about harmful effects of violence and the benefits of a relationship based on equality. The COMBAT organise public meetings, in churches, during community festivals, association or group meetings and through radio programs. They use various strategies such as role plays to introduce the topic they will be discussing. Once the presentation is made, opportunity is given for the audience to ask questions.

The work of COMBAT is linked to state agencies through a referral system. COMBAT may accompany a victim to report to an agency e.g. GHS, police, DSW, or CHRAJ to support the victim who is engaging with the service for the first time and might be nervous about doing so. The Gender Centre’s experience from prior implementation of the RRS has shown that when a victim of violence seeks outside support, it is important to have other people to be with them, such as family and friends [7]. The COMBAT follows up on cases that have been referred to service provider and the victim herself. Considering the social context within which COMBAT operates, it is stressed that there are certain issues that can only be dealt with by law and that should not be compromised by mediation; but at the same time, COMBAT are taught to respect the victim’s choice and not to force the victim to do something they do not want to do.

#### Post-training support and monitoring

Refresher training for COMBAT and staff of the selected state agencies is provided one year after the initial training while subsequent trainings on counselling, family laws and conflict resolution are carried out every three months. Each COMBAT is supported by an animator who supports the team in system documentation. The animators have a biweekly meeting with COMBAT and are responsible for submitting monthly reports to the Gender Centre project staff. Staff from the Gender Centre are assigned to provide technical support to the COMBAT. This involves bi-monthly meetings with the teams and participating in some of the community sensitisations. In addition, the COMBAT have telephone access to the Gender Centre staff to clarify any issues they may have while on the field. The work of the COMBAT is monitored through quarterly community reflection meetings. The objective of the community reflection sessions is to provide an opportunity for the community members to give their views on the implementation of the RRS and the work of the COMBAT.

#### Study outcomes

The main study outcome is past year incidence of IPV (i.e. perpetration of physical and/or sexual IPV for men and experiences for women) which is assessed with the quantitative survey. The questions and response scale used to asses past 12 months IPV experience or perpetration in our population are listed in Table 1. Secondary outcome indicators in our survey address gender attitudes (women’s subordination, Tolerance of VAW, VAW perpetration, and controlling behaviours), social stigma for the victims of VAW, institutional response to VAW, relevant laws on VAW, women and societal responses to VAW and mental health issues). We report of no changes to the trial outcomes after the trial commenced.

**Table 1.** Tools for measuring past year IPV experience or perpetration among adult women and men in 4 districts of the central region of Ghana.

*Response scale – If yes, how often did this happen? Never Once, More than once*	
IPV EXPERIENCE BY WOMEN	IPV PERPETRATION BY MEN
**Physical violence by intimate partner**	**Physical violence against intimate partner**
● Was slapped or had something thrown at her that could hurt her	**●** Slapped or thrown something at her that could hurt her
● Was pushed or shoved	**●** Pushed or shoved her
● Was hit with fist or something else that could hurt.	**●** Hit her with fist or something else that could hurt.
● Was kicked, dragged, beaten, choked or burnt	**●** Kicked, dragged beat, choked or burnt her
● Perpetrator threatened to use or actually used a weapons against her	**●** Threatened to use or actually used a weapons against her
**Sexual violence by an intimate partner**	**Sexual violence against an intimate partner**
● Was physically forced to have sex when she did not want	**●** Physically forced to have sex when she did not want
● Had sex when she did not want to because she was afraid of what the partner might do	**●** Had sex when she did not want to because she was afraid of what I might do
● Was forced to do something sexual that she found degrading or humiliating	**●** Forced her to do something sexual that she found degrading or humiliating
● Was forced to watch pornography	**●** Forced her to watch pornography
**Emotional abuse by an intimate partner**	**Emotional abuse against an intimate partner**
● Was insulted or made to feel bad about yourself	**●** Insulted or made her to feel bad about herself
● Was belittled or humiliated in front of other people	**●** Belittled or humiliated in front her of other people
● Perpetrator had done things to scare or intimidate her on purpose (e.g. by yelling or smashing things)	**●** Did things to scare or intimidate her on purpose (e.g. by yelling or smashing things)
● Perpetrator had threatened to hurt her or someone she cared about	**●** Threatened to hurt her or someone she cared about
● Perpetrator boasted about or brought home girlfriend	● Boasted about or brought home girlfriend
**Economic abuse by an intimate partner**	**Economic abuse against an intimate partner**
● Prohibited her from getting a job, going to work, trading or earning money	**●** Prohibited a partner from getting a job, going to work, trading or earning money
● Taken her earnings against her will	**●** Taken a partner’s earnings against her will
● Kept money from her earnings for alcohol, tobacco or other things when he knew she was finding it hard to afford the household expenses	**●** Kept money from your earnings for alcohol, tobacco or other things for yourself when you knew your partner was finding it hard to afford the household expenses

## Study tools

Both qualitative and quantitative measures are used in this trial to evaluate multiple perspectives, contextualise information, and develop a more complete understanding of VAW and IPV in the communities. Quantitative data include standard measures developed by the World Health Organisation for its multi-country study on VAW [6]. These questionnaires have been tested in South Africa over many years in different populations and most recently used in the pilot of Stepping Stones and Creating Futures in South Africa (SS CF pilot) [14,15]. Our trial questionnaires were initially translated into local dialects (Fante and Twi) by an independent consultant and then edited by bi-lingual members of the project team at the University of Ghana. The revised translations were then independently back translated by another consultant who had not seen the English version of the questionnaire. The project team then used a consensus building translation approach to finalise the translated questionnaire. This involved discussing and resolution of discrepancies together with data collectors at the baseline training.

The qualitative component is intended to provide in-depth understanding of the effects of the COMBAT intervention, if any, at individual, community as well as institutional levels. Qualitative data collection was done at baseline and will be repeated at midpoint and the endpoint of the intervention in both intervention and control communities. Focus group discussions (FGDs) are designed to capture perceptions of the community on VAW as well as the components of the proposed intervention. Community members are engaged for the examination of group norms and acts regarding VAW in various contexts. FGD participants may be IPV victims, perpetrators or neither. They may or may not have also been involved in the baseline survey. FGDs are organised separately for women and men to allow for expression of gendered views within a comfortable and convenient environment without any apprehension. All FGDs are gender-matched, where male and female research assistant’s moderate male FGDs and female FGDs, respectively. COMBAT Members’ programmatic perspectives are also captured using FGDs to understand the activities regarding awareness creation on gender-based violence as well as support provided to victims of violence. In-depth interviews (IDIs) are conducted among community members who have had previous VAW experiences (identified from the baseline quantitative survey). Focusing on such individuals allow us to capture-specific VAW experiences in these communities and examine how these experiences highlight the knowledge and understanding of the negative impact of VAW as a social issue as well as actions taken. We plan to engage the same community members at the various data collection points to not only capture changes in knowledge levels, but also to assess the recurrence of VAW acts and individual responses to such recurrence.

Key Informant Interviews (KIIs) are conducted with critical stakeholders and decision-makers in institutions mandated to address VAW cases within the various communities. These key personnel are interviewed for their perspectives on relevance, effectiveness, strengths and weaknesses of the program and the current response mechanisms, and their recommendations for improvement. The interviews also provide useful information on performance of institutional structures, best practices, and collaborations and partnership that are in place to ensure effectiveness of the intervention delivery and sustainability. We further review institutional documents of the critical state agencies mandated to handle VAW issues. Institutional documents used for capturing reports of VAW cases as well as actions taken on such reports are sourced and reviewed from both the intervention and control sites. Details of all cases reported between, 2015 and 2018 will be extracted including number of cases reported, who is reporting information, follow-up on cases reported, recurrence of VAW, etc. Data gathered through document reviews will be triangulated with information from the KIIs, IDIs and FGDs.

## Data collection

### Pre-data collection activities

Pre-data collection fieldwork was carried out in three phases; mapping of study area, pre-testing of data collection instruments, and community mobilisation. Mapping of localities was facilitated by Enumeration Area data obtained from the Ghana Statistical service. Listing of all households in the selected localities was undertaken by trained research assistants and this was used for the computerised random selection of participating households. Cognitive testing of questionnaires was also undertaken to ensure understandability and check appropriateness of responses, including Likert scale options. The questionnaires were pre-tested in a population with the same characteristics as the study population prior to main data collection period. Pre-testing enabled us to test the clarity and suitability of the questions and the results led us to make appropriate changes to the tool. Prior to baseline data collection, community mobilisation, led by the Gender Centre was done to introduce the research in the study sites and to facilitate the recruitment of participants.

## Ethics

Ethical approval for this trial was obtained from the Institutional Review Board at the Noguchi Memorial Institute for Medical Research at the University of Ghana (# 006/15–16) and the South African Medical Research Council’s Ethics Committee (EC031-9/2015). Our research procedure agrees with the ethical principles outlined by the World Medical Association Declaration of Helsinki [16] and the Belmont report [17]. Our trial has been designed to ensure that the project does not expose participants to more than minimal risk (adverse consequences i.e. emotional or psychological harm). We also recognise that complete privacy is essential for ensuring the security of the respondent and the interviewer. Asking about or reporting violence, especially in households where the perpetrator may be present at the time of interview, carries the risk of further violence. Accordingly, our interviewers were provided specific training for implementing the domestic violence module to enable the field staff to collect violence data in a secure, confidential, and ethical manner. The same approach will be followed during the other data collection phases of the trial.

### Informed consent

All research participants provided written informed consent before enrolling in the trial. Participants were informed of the purpose of the trial, trial procedures, potential risks and benefits of the trial and with their rights as participants explained to them. The participant information and consent document are written in simple English, however, to enhance understanding, a researcher is present throughout the informed consent process to clarify any questions the participants were not clear about. Those consenting, signed (or placed a thumb print on) an informed consent form before participating in trial procedures.

### Confidentiality and anonymity

All participants were assured that the information provided will be handled confidentially and that findings will be reported with complete anonymity. This trial uses interviewer –administered tablets for collecting the quantitative data. Electronic handheld devices have been used by the investigators in previous VAW research with young and adult men and women in Ghana, South Africa and elsewhere with great success with both literate and illiterate people [18,19]. Use of electronic hand-held tablets in this trial ensures anonymity of information given by participants as they do not write their names in the tablet. Participants were given unique study codes that are not linkable to their names and thus their responses cannot be traced back to them. Whilst the trial includes questions about engaging in illegal domestic violence activities, we do not collect adequate information about any act in the questionnaire to enable a prosecution of any crime.

### Harm due to trial participation

As part of the trial procedures, we collect information on risks related to the trial. While it is probable that there may be deaths of participants during the trial period, it is highly unlikely for these deaths to be trial related as we will not be administering medication to participants. Notwithstanding this, if a death of the participant occurs, we will promptly investigate on the circumstances and cause of death and a report will be sent to the ethics committees whether the death is found to be trial related or not. This research includes many sensitive topics. It has been well established that there is a potential for harm to participants in research on gender-based violence. The most important concerns relate to the risk of re-victimisation of women who will participate in this trial; and the risk of psychological distress to participants through being asked to re-remember traumatic events. The WHO guidance on safety in conduct of gender-based violence research recommend that the nature of the research be concealed from non-interviewees [20]. Research from diverse global settings has shown that women victims of violence are saddened by talking about their exposure to violence but overwhelmingly they welcome a chance to talk and many describe the research interview as a life-changing occurrence [13,19–22]. Additional research has shown that if these guidelines are followed, there are minimal risks attached to survey research on gender-based violence [15,20]. We have strictly adhered to this guidance in this trial, however, in cases where participants demonstrate distress or report being emotionally impacted by the research questions or intervention, they will be provided with referrals to professional services available in their communities.

### Participant compensation

All participants receive a cash compensation of 10 GHC (~ $3USD) for participating in the trial. No incentive is provided for intervention participation and this is explained to the participants in the intervention arm prior to their enrolment in the study. We are comfortable with this participant compensation approach as research elsewhere has shown that financial incentives alone are not enough to prevent violent experiences or practices [14,15] and so we anticipate that we can meaningfully incentivise both study arms without interfering with the study main outcome.

## Data analysis

The main trial analysis will be done by intention to treat. Thus, for intervention districts, data for all respondents will be included whether they report contact with the intervention or not. Any participant who responds affirmatively to any one of the five questions on physical IPV or three questions on sexual IPV in the past 12 months at end line data collection point will be deemed to be an incident case of IPV victimisation (if woman) or perpetration (if man). IPV incidence will be compared between the study arms. Generalised Linear Mixed model for binary outcome (logistic regression) will be used to compare women’s past year experience or men’s perpetration of IPV between the two arms at end-point, adjusting for baseline prevalence. All qualitative data will be coded, and thematic approach will be used to evaluate the impact of the interventions implemented within the communities.

## Discussion

Intimate partner violence (IPV) is one of the most common forms of violence against women (VAW) and its prevention requires comprehensive intervention strategies. Our Rural Response System intervention uses Community-Based Action Teams (COMBAT) to sensitise communities about IPV. To share best practices learned in conducting this trial, we discuss some challenges encountered during the study implementation as well as mitigation strategies adopted to resolve these issues. To begin with, the intervention was informed by the theory of change logic model which is an established framework using a community-based participatory approach for reducing VAW [23]. The unique nature of this intervention required that we conduct baseline interviews and FGDs with COMBAT volunteers soon after they are selected before training. Engaging COMBAT at baseline, midline, and end time points would allow for the research team to capture changes in COMBAT members’ knowledge in relation to VAW as a social issue and learn about COMBAT members’ experiences with the implementation of the intervention, as well as explore their perceptions of the community’s response to the intervention. Assessing the change in knowledge and growth of the COMBAT volunteers is fundamental as COMBAT are the main vehicle through which the intervention will be delivered. The challenge in documenting COMBAT baseline knowledge, attitudes and perceptions on VAW presented in the form of difficulty in getting all COMBAT volunteers to arrive at their training site on time so that the baseline surveys and FDGs can be done before they start their training. This was a daunting task since majority of COMBAT had no mobile phones, come from different communities in the selected districts, and are also volunteers hence might be unwilling to leave their regular work at certain times. Our mitigation approach was to contact focal persons in each district (i.e. the COMBAT animator/leader) so that they can organise the COMBAT locally and get them to arrive at the training facility in one group.

Organisation of FDGs with community members was challenging in communities where locals migrate for work during certain times of the year. Thus, project supervisors coordinated with key informants in the community to find suitable times and venues for all planned FDGs. Review of VAW record at state institutions proved tedious on occasion as some intuitions did not have up-to date records or keep soft copies of reported cases. Project supervisors therefore engaged with institutional directors/heads prior to data collection to ascertain what information was available and in what format it was kept. Availability of respondents (especially men) during the working day was at times problematic and field workers had to schedule and reschedule interviews on weekends, late afternoons and evenings to meet the respondent’s availability. It may be important to note that this situation was only encountered in communities where men predominantly migrated to find work in neighbouring communities. Although both men and women in farming communities were away on the farm most mornings and afternoons, they were usually willing to wait at home for field staff on scheduled appointments, e.g. very early in the mornings.

This trial has a few limitations that we sought to lessen. Where possible measures to counteract factors that may impact interpretation of study results have been considered *a-priori*. For instance, to addresses analytical challenges that will present from incomplete surveys, we have oversampled the study population by 15%. To check errors associated with instrumentation effects, the same field project staff will administer questionnaires at baseline and again at post-intervention assessment in each district. Regarding testing effects, we do not anticipate that taking part in the baseline survey might influence the treatment effects, or that it might bias the impact-assessment responses. While we do anticipate that there might be movement and mingling of study participants between treatment arms during the trial, questions pertaining to exposure/interaction with intervention and/or staff will be included at post-assessment for both treatment groups to account for any contamination. In addition, other GBV related advocacy/intervention activities on-going at the time of intervention delivery will be documented and participant contact with these will be assessed at post intervention. It is always possible with behavior research that there may be under or over-reporting and this could be differential at end line by study arm. Unfortunately, we are not able to prevent this.

In conclusion, the increasing prevalence of intimate partner violence in Ghana over the last three decades point to fact that intervening at the individual level may not be the optimal way to prevent and/or reduce the occurrence of IPV. Current evidence has demonstrated the importance of considering the context of intimate violence, including the type of relationship in which IPV occurs, the relevant gender roles and social norms, among other factors [15,24]. The use of mixed methods in this trial provides stronger evidence through convergence and corroboration of findings, which gives far more insight and understanding on IPV than when a single method or measurement is used. This trial therefore provides a robust research foundation that will effectively inform future interventions on IPV prevention, especially in a low-middle income setting.

## Paper context

We describe a mixed method impact assessment for an on-going multifaceted intervention known as the rural response system in the Central Region of Ghana. This intervention uses Community-Based Action Teams to increase awareness about intimate partner violence, whilst promoting a community-focused response mechanism. Findings from our trial will provide a robust science-driven foundation that will effectively inform future interventions on intimate partner violence prevention, particularly in resource-poor settings.

## Appendix 1: Intervention protocol – training programme on violence against women

**Table:**

Day 1	Day 2	Day 3	Day 4	Day 5
Introductions	Recap Day One	Recap Day Two	Recap Day Three	Recap Day Four
Hopes and Fears	**Explaining violence**	**Myths About Violence**	**Understanding Violence**	**Responding to victims of violence**
Objectives of Training	Gender Reflection Exercises (as part of root causes)	Messaging and stereotypes	Understanding why women stay in abusive relationships	Awareness at personal level when responding to violence
Review of Agenda	Baby Exercise	Sex role stereotypes	Cycle of Violence	Assessment of Victims
Parking Lot and Selection of Prefects	Gender Messaging	Expectations in different types of relationships	Isolation as a key factor in violent relationships	What help does an abused woman want
**B R E A K**				
**Defining Violence**	**Gender Roles/Biology vs. Gender**	**Types of Violence**	**Facts about Relationship Violence**	What you can do to help an abused woman
Why is violence against women an issue?	Stepping out of Roles	Physical violence	Equality as a factor in relationships	Best Practice: Communication and Listening skills
Defining violence UN definition	Social Construction of gender summary	Psychological violence	Options for an abused woman	Confidentiality
Important Ingredients of UN Definition	**Root causes of Violence**	Sexual violence		Dealing with abused children
Importance of Definitions	Patriarchy	Economic Abuse		Evaluation
Definition of violence from Ghana research	Power and Control	Facts on violence from Ghana research		
	Social and Structural Influences supporting violence	Impacts of Violence		
**C L O S I N G**				

## Appendix 2: Counselling Training Programme

This is a 4-day counselling training to equip community-based action teams with basic counselling skills to enable them to provide basic services in an appropriate and sensitive way to victims of domestic violence in rural communities

**Table:**

Day 1	Day 2	Day 3	Day 4
Overview of domestic violence and its legal, medical and psychological impact	Practical issues the abused woman brings into counselling	Identifying Community resources & Referral	Some Ethical principles
Introduction to counselling	Counselling skills II (reflecting and summarising)	Safety Planning	Role play
**B R E A K**			
Counselling skills I (listening & Clarifying)	Role play of counselling skills	Role play of referral	
Role Play of counselling skills	Assessment	Qualities of a good counsellor	
Stages in counselling	Role play of counselling situation	Fears of the Counsellor	
Role play of stages one & two using listening skills		Fears of the Client in the counselling relationship	
**C L O S I N G**			

## References

[1] Campbell JC. Health consequences of intimate partner violence. Lancet. 2002;359:1331–14. 11965295 10.1016/S0140-6736(02)08336-8

[2] Coker AL, Davis KE, Arias I, et al Physical and mental health effects of intimate partner violence for men and women. Am J Prev Med. 2002;23:260–268. 12406480 10.1016/s0749-3797(02)00514-7

[3] Bonomi AE, Thompson RS, Anderson M, et al Intimate partner violence and women’s physical, mental, and social functioning. Am J Prev Med. 2006;30:458–466. 16704938 10.1016/j.amepre.2006.01.015

[4] Jwekes R Intimate partner violence: causes and prevention. Lancet. 2002;359:1423–1429. 11978358 10.1016/S0140-6736(02)08357-5

[5] Kovacs RJ The macro-level drivers of intimate partner violence: new evidence from a multilevel dataset. Glob Public Health. 2017 5 4; 1–13. DOI:10.1080/17441692.2017.1317010. 28468526

[6] Garcia-Moreno C, Jansen HA, Ellsberg M, et al WHO multi-country study on women‘s health and domestic violence against women study team. Prevalence of intimate partner violence: findings from the WHO multi-country study on women‘s health and domestic violence. Lancet. 2006 10 7;368:1260–1269. 17027732 10.1016/S0140-6736(06)69523-8

[7] Gender Studies and Human Rights Documentation Centre, Breaking the Silence and Challenging the Mythos of Violence against Women and Children in Ghana Report of a national study on violence. Edited by Dorcas Coker-Appiah and Kathy Cusack. Gender and Human Rights Documentation Centre, 1998.

[8] Ghana Statistical Service (GSS), Ghana Health Service (GHS), and ICF Macro (2009). Ghana Demographic and Health Survey 2008. Accra, Ghana: GSS, GHS, and ICF Macro.

[9] UN Women Violence against women prevalence data: surveys by country. [cited 2017 929]. Available from: http://www.endvawnow.org/uploads/browser/files/vaw_prevalence_matrix_15april_2011.pdf

[10] Ghana AIDS Commission (GAC) The HIV and AIDS programme of work 2009. Accra, Ghana: GAC; 2009b.

[11] Ghana Statistical Service (GSS) Population and housing census, regional analytical report; central region. Ghana: Ghana Statistical Service; 2010.

[12] Hayes R, Bennett S Simple sample size calculation for cluster randomised trials. Int J Epidemiol. 1999;28:319–326. 10342698 10.1093/ije/28.2.319

[13] Pence E, Paymar M Education groups for men who batter: the Duluth model. New York, NY: Springer Publishing Company; 1993.

[14] Jewkes R, Gibbs A, Jama-Shai N, et al Stepping Stones and Creating Futures intervention: shortened interrupted time series evaluation of a behavioural and structural health promotion and violence prevention intervention for young people in informal settlements in Durban, South Africa. BMC Public Health. 2014;14:1325. 25544716 10.1186/1471-2458-14-1325 PMC4320600

[15] Gibbs A, Washington L, Willan S, et al The stepping stones and creating futures intervention to prevent intimate partner violence and HIV-risk behaviours in Durban, South Africa: study protocol for a cluster randomised control trial, and baseline characteristics. BMC Public Health. 2017;17:336. 28427380 10.1186/s12889-017-4223-x PMC5397780

[16] World Medical Association declaration of Helsinki Ethical Principles for Medical Research Involving Human Subjects. [cited 2017 929]. Available from: https://www.wma.net/policies-post/wma-declaration-of-helsinki-ethical-principles-for-medical-research-involving-human-subjects/ 10.1191/0969733002ne486xx 16010903

[17] The Belmont report. Office of the secretary, ethical principles and guidelines for the protection of human subjects of research. The National Commission for the Protection of Human Subjects of Biomedical and Behavioural Research. [cited 2017 929]. Available from: http://www.hhs.gov/ohrp/humansubjects/guidance/belmont.html 25951677

[18] Jewkes R, Sikweyiya Y, Morrell R, et al Why, when, and how men rape: understanding rape perpetration in South Africa. SA Crime Quarterly. 2010; 34: 23–31.

[19] Sikweyiya Y, Jewkes R, Morrell R Talking about Rape: men’s responses to questions about rape in a research environment in South Africa. Agenda. 2007;74:48–57.

[20] Putting women first: ethical and safety recommendations for research on domestic violence against women. Geneva, Switzerland: World Health Organization, 2001. [cited 2017 929]. Available from: http://www.who.int/gender/violence/womenfirtseng.pdf

[21] Sikweyiya Y, Jewkes R Perceptions about safety and risks in gender-based violence research: implications for the ethics review process. Cult Health Sex. 2011;13:1091–1102. [cited 2017 9 29] 21824018 10.1080/13691058.2011.604429

[22] Jewkes R, Watts C, Abrahams N, et al Ethical and methodological issues in conducting research on gender-based violence in Southern Africa. Reprod Health Matters. 2000;8:93–103. 11424273 10.1016/s0968-8080(00)90010-7

[23] Weiss CH Nothing as practical as good theory: exploring theory-based evaluation for comprehensive community initiatives for children and families. (Connell, J, Kubisch, A, Schorr, L, and Weiss, C. (Eds.) ‘New Approaches to Evaluating Community Initiatives’ ed.). Washington, DC: Aspen Institute, 1995 65

[24] McHugh MC, Frieze IH Intimate partner violence, new directions. Ann NY Acad Sci. 2006;1087:121–141. 17189502 10.1196/annals.1385.011

